# Sentinel lymph node biopsy in melanoma: Our 8-year clinical experience in a single French institute (2002–2009)

**DOI:** 10.1186/1471-5945-12-21

**Published:** 2012-12-10

**Authors:** Caroline Biver-Dalle, Eve Puzenat, Marc Puyraveau, Delphine Delroeux, Hatem Boulahdour, Frances Sheppard, Fabien Pelletier, Philippe Humbert, François Aubin

**Affiliations:** 1 Department of Dermatology, Besançon University Hospital, Besançon, France; 2 Clinical Methodology Center, Besançon University Hospital, Besançon, France; 3 Department of Digestive Surgery, Besançon University Hospital, Besançon, France; 4 Department of Nuclear Medicine, Besançon University Hospital, Besançon, France; 5 University of Franche Comté, UMR1098, SFR FED4234, Besançon, France; 6 University of Franche Comté, EA3181, SFR FED4234, Besançon, France; 7 Service de Dermatologie, 2 Place Saint-Jacques, 25030, Besançon, cedex, France

**Keywords:** Melanoma, Sentinel lymph node

## Abstract

**Background:**

Since the introduction of sentinel lymph node biopsy (SLNB), its use as a standard of care for patients with clinically node-negative cutaneous melanoma remains controversial. We wished to evaluate our experience of SLNB for melanoma.

**Methods:**

A single center observational cohort of 203 melanoma patients with a primary cutaneous melanoma (tumour thickness > 1 mm) and without clinical evidence of metastasis was investigated from 2002 to 2009. Head and neck melanoma were excluded. SLN was identified following preoperative lymphoscintigraphy and intraoperative gamma probe interrogation.

**Results:**

The SLN identification rate was 97%. The SLN was tumor positive in 44 patients (22%). Positive SLN was significantly associated with primary tumor thickness and microscopic ulceration. The median follow-up was 39.5 (5–97) months. Disease progression was significantly more frequent in SLN positive patients (32% vs 13%, p = 0.002). Five-year DFS and OS of the entire cohort were 79.6% and 84.6%, respectively, with a statistical significant difference between SLN positive (58.7% and 69.7%) and SLN negative (85% and 90.3%) patients (p = 0.0006 and p = 0.0096 respectively). Postoperative complications after SLNB were observed in 12% of patients.

**Conclusion:**

Our data confirm previous studies and support the clinical usefulness of SLNB as a reliable and accurate staging method in patients with cutaneous melanoma. However, the benefit of additional CLND in patients with positive SLN remains to be demonstrated.

## Background

Since its introduction in 1992 [1], the role of sentinel lymph node biopsy (SLNB) in melanoma care remains controversial and is not included in most guidelines for the management of melanoma in Europe [2]. Its main short term aim is the early identification of patients with occult nodal metastasis, known as micrometastasis, who might benefit from complete lymph node dissection (CLND). The long term aim is to provide a more accurate basis for formulating a prognosis than do standard demographic and histopathological factors. Furthermore, the presence or absence of micrometastases in the sentinel lymph node (SLN) is critical to both accurate AJCC staging^2^ and decisions regarding adjuvant therapy and follow-up regimens. The final version of melanoma staging and classification takes into account the results of SLNB [3]. A Cox multivariate analysis of 3,307 stage III patients demonstrated that 5-year survival rates ranged from 70% for patients with micrometastasis (T1-T4N1aM0) to 39% for patients with T1-T4N3M0 CM. However, according to the Multicenter Selective Lymphadenectomy Trial (MSLT) [4], there was no significant difference in disease-specific survival between patients with lymphatic mapping by SLNB (and immediate CLND) and patients with nodal observation. Other retrospective studies have shown similar results and the influence of SLNB and CLND on long term patient survival as well as its therapeutic role are still debated [5].

Despite the fact that SLNB is widely used in France [2], there are no French studies reporting the experience of SLNB. We present our 8-year consecutive clinical experience of performing SLNB for CM. We evaluated the outcome of patients in terms of disease progression and mortality based on the SLNB result.

## Methods

### Patients

SLNB has been performed at Besançon University Hospital since 2000. Patients who had undergone this technique in the first two years were excluded to allow the medical team to gain experience in guaranteeing reproducibility and reliability of the results [4]. Only patients with a primary cutaneous melanoma (tumour thickness > 1 mm) and without clinical evidence of metastasis who underwent SLNB between January 2002 and December 2009 were included. Furthermore, patients with head and neck CM were also excluded because of the complexity of lymphatic drainage, multi-site drainage and the high number of false negatives [6,7]. Since this retrospective study was conducted in France, it was not eligible for submission to our research ethics committee. Patients were selected and each clinical file obtained from the Cancer Registry of the Besançon University Hospital (authorization from the Privacy and Data Protection National Agency, CNIL number 903417) was re-examined and the following data were collected: epidemiological criteria (sex, age), histological criteria, clinical features, SLN status (positive or negative), results of CLND and evolution criteria (relapse and survival). The epidemiological and histological data were collected from the Cancer Registry, whereas the evolution criteria were gathered from the hospital clinical files or by writing to family practitioners.

### SLNB procedure

After information and written consent, preoperative lymphoscintigraphy was performed in all patients by injecting 1 mL of technetium Tc 99 m-labeled sulfur colloid intradermally around the periphery of the primary lesion or biopsy scar in 4-quadrant fashion. Using a gamma camera with a low-energy, high resolution collimator, dynamic and static images were obtained, beginning 15 minutes after injection and continuing every 30 minutes thereafter, until the SLNs were visualized. Surgery took place the following day. A hand-held gamma probe was used to localize the SLN transcutaneously. The SLN was identified intra-operatively using a gamma probe. After SLN harvesting, the radioactive count was measured ex vivo using the gamma probe. Echelon nodes were then harvested if they had a count ≥ 10% of the SLN. The background count of the lymph node basin was then measured to ensure that no further radioactive nodes remained. In addition to preoperative lymphoscintigraphy, 9 patients received an on-table injection of patent Blue V dye (Laboratoire Guerbet, Aulnay-sous-Bois, France) around the biopsy scar. Following completion of SLN dissection, the maximal counts per second *in vivo* and *ex vivo* were recorded to verify that no areas of increased radioactivity remained.

### Histopathologic evaluation

Pathological analysis of SLN involved an initial bisection of the node along its hilum after fixation. Then, from each side of the SLN, five serial step sections of 4 mm were cut with 50 mm intervals between different numbers of sections. Finally, all sections were stained with hematoxylin and eosin. All slides were examined histologically, and if melanoma cells were detected immunohistochemistry (S100, HMB45 and MelanA) was then performed for confirmation.

### Surgical and adjuvant therapy

Patients with positive SLNs were advised to have CLND of the regional basin. According to French guidelines, all patients with primary CM larger than or equal to 1.5 mm in thickness as well as patients with positive SLN and positive CLND and patients with high-risk primary melanoma (tumor thickness > 4 mm and ulceration) were considered for adjuvant interferon alpha therapy, low-doses and high doses of interferon, respectively. Demographic, clinical and histological characteristics of patients together with primary CM, SLNB and CLND pathological reports, and the lymphoscintigraphy imaging file and surgery report were collected.

### Follow-up

Patients were followed up in an outpatient setting by clinical examination one week postoperatively and then on a six monthly basis for the first three years and every year for the next 5 years. In addition, ultrasound analysis of regional lymph nodes was performed in patients with positive SLN. Tumor progression and survival status were gathered from the hospital clinical files or by writing to family practitioners and the observations were censored on December 31st 2010.

### Statistical analysis

Statistical analysis was based on chi squared analysis or the exact Fisher test for qualitative data and based on Student test for quantitative data, Kaplan-Meier survival curves and log rank analysis. The significance level was determined at p less than 0.05. The analyses were performed with SAS software, version 9.2 (Sas Institute, Inc, Cary, NC).

## Results

From January 2002 to December 2009, 203 patients (100 men and 103 women) with melanoma thickness superior to 1 mm underwent SLNB. The mean age was 56 +/− 16 (16 to 86). Clinical and histological characteristics are shown in Table 1.

**Table 1:** SLN: sentinel lymph node

	**Entire cohort**	**Patients with positive SLN**	**Patients with negative SLN**	**p**
**Number**	203*	44/197* (22%)	153/197* (78%)	
**Sex**				0.2706
**Men**	100 (49.3%)	18 (41%)	76 (50%)	
**Women**	103 (50.7%)	26 (59%)	77 (50%)	
**Mean age (+/− SD)**	55.8 +/−15.6	52.0 +/−17.4	56.6 +/−15.0	0.0864
**Localization**				0.1483
Trunk	74 (36.5%)	17 (38.6%)	54 (35.3%)	
Upper limb	39 (19.2%)	3 (6.8%)	33 (21.6%)	
Lower limb	63 (31.0%)	16 (36.4%)	47 (30.7%)	
Hands and feet	27 (13.3%)	8 (18.2%)	19 (12.4%)	
**Type**				0.6978
Superficial	115 (56.6%)	25 (56.8%)	86 (56.2%)	
Nodular	39 (19.2%)	6 (13.6%)	31 (20.3%)	
Acral	17 (8.4%)	4 (9.1%)	13 (8.5%)	
Other	32 (15.8%)	9 (20.5%)	23 (15.0%)	
**Mean tumor thickness (range)**	1.88 (0.4 - 10.1)	2.8 (1.2 - 10)	1.6 (0.4 - 10.1)	0.0289
**T stage**[2]				0.0172
T1	10 (4.9%)	0	10 (6.5%)	
T2	101 (49.7%)	15 (34.1%)	83 (54.3%)	
T3	55 (27.1%)	17 (38.6%)	36 (23.5%)	
T4	33 (16.3%)	11 (25.0%)	21 (13.7%)	
Incalculable^1^	4 (2.0%)	1 (2.3%)	3 (2.0%)	
**Clark level**				0.3677
I	1 (0.5%)	0	1 (0.7%)	
II	10 (4.9%)	2 (4.5%)	8 (5.2%)	
III	56 (27.6%)	9 (20.5%)	46 (30.1%)	
IV	100 (49.3%)	26 (59.1%)	70 (45.7%)	
V	12 (5.9%)	4 (9.1%)	7 (4.6%)	
Unknown	24 (11.8%)	3 (6.8%)	21 (13.7%)	
**Ulceration**				0.0080
Yes	60 (29.6%)	21 (47.7%)	37 (24.2%)	
No	131 (64.5%)	20 (45.5%)	107 (69.9%)	
Unknown	12 (5.9%)	3 (6.8%)	9 (5.9%)	

NS: not significant.

*6 complete or partial failures (lymphoscintigraphy and surgery).

### Sentinel lymph node biopsy

In all but 6 patients (3%), SLN was identified. A complete failure (absence of reliable scintigraphic imaging and surgical localization) was observed in two patients with truncal melanoma. A surgical failure (i.e. absence of SLN sample) despite scintigraphic localization was reported in two patients with upper limb melanoma. SLN localization and excision could not be carried out in two patients because there was no percutaneous radioactivity on the incision area. The same level of radioactivity was localized in several axillary lymph nodes in one patient, and CLND was then performed and was negative. The axillary tissue harvested during SLNB procedure did not contain any lymph nodes in one patient. These 6 patients had not undergone blue dye marking.

The SLN identification rate was 97%. The mean number of SLN harvested was 1.5 +/− 1. Only one SLN was harvested in 67% of cases. Nodal basin included unilateral axilla (87 cases), unilateral groin (85 cases), bilateral axillae (11 cases), bilateral groins (1 case), popliteal fossa (2 cases) and epitrochlear (0 case). The drainage area for limb melanomas was always homolateral (data non shown). Drainage to multiple node fields was present in 37 cases (18%) and most of them (57%) originated from dorsal melanomas. Of patients with trunk melanoma, 13 demonstrated interval nodes (17%). Of patients with limbs melanoma, we observed interval nodes, including popliteal (9 patients, 14%), and epitrochlear nodes (2 patients, 5%). Of these 24 patients with interval nodes, 11 had samples taken surgically, demonstrating a melanoma micrometastasis in three patients.

Of the 197 patients in whom a SLN was identified, 44 (22%) were tumor positive. We observed a statistically significant difference between positive and negative SLN patients for tumor thickness (p = 0.0289), the presence of ulceration (p = 0.008), and T stage (p = 0.0172) in primary CM.

### Complete lymph node dissection and adjuvant therapy

Of the patients with positive SLN, 95% (42 of 44 patients) underwent additional CLND. One patient had a popliteal positive SLN and refused further surgical intervention and one patient had contraindications for radical lymphadenectomy. None of them relapsed. Eleven patients (25%) had further pathologically positive lymph nodes. Of the patients with positive SLN, 29% were treated with interferon alpha. Eleven patients (7%) with negative SLN but with a high-risk primary melanoma (tumor thickness > 4 mm and ulceration) were treated with interferon.

### Recurrence

Patients were followed for a median duration of 39.5 months (range: 5 – 97). Fifteen patients (5 SLN positive patients and 10 SLN negative) were lost to follow-up.

Thirty-four patients (17%) relapsed. Recurrences were significantly more frequent (p = 0.002) in SLN positive patients (32%) than in SLN negative patients (13%). The median time for recurrence in our cohort was 12 months (range: 0–58 months), with no significant difference between SLN positive and negative patients (15.2 +/− 15.6 months versus 17.4 +/− 16.6 months, respectively, p = 0.7107). The site of initial recurrence is shown in Table 2. There was no significant difference in the type of recurrence between positive and negative SLN patients. The result of CLND in SLN positive patients did not lead to any significant differences in terms of relapse, type of relapse or death (data not shown).

**Table 2:** Site of initial recurrence by sentinel lymph node status

	**Number all patients**	**SLN positive patients**	**SLN negative patients**	**p**
**SLN status**	197	44 (22%)	153 (78%)	
**Recurrence:**				
**Yes**	34 (17%)	14 (32%)	20 (13%)	0.002
**No**	163 (83%)	30 (68%)	133 (87%)	
Site of initial recurrence*:				
**Local/in transit**	17 (50%)	9 (64%)	8 (40%)	NS
**Regional lymph node**	16 (47%)	6 (43%)	10 (50%)	NS
**Distant metastatic**	20 (59%)	8 (57%)	14 (70%)	NS

*Some patients may present multiple sites of recurrence.

SLN: sentinel lymph node.

NS: not significant.

The percentage of false-negative patients in our cohort was 6.5%, as 10 of the 153 SLN negative patients developed regional lymph node relapse. Sensitivity, specificity and the positive and negative predictive values of the SLN status (Table 3) in terms of recurrence were 41%, 81%, 32% and 87% respectively. Sensitivity, specificity and the positive and negative predictive values of the SLN status in terms of mortality were 39%, 80%, 20% and 91% respectively.

**Table 3:** SLN status sensitivity, specificity, positive predictive value (PPV) and negative predictive value (NPV) in terms of recurrence and mortality

	**SLN +**	**SLN -**	**Number of patients**
**Recurrence**	14 (TP)	20 (FN)	34
**No recurrence**	30 (FP)	133 (TN)	163
**Death**	9 (TP)	14 (FN)	23
**Living**	35 (FP)	139 (TN)	174
**Number of patients**	44	153	197

TP: true-positive; TN: true-negative; FP: false positive; FN: false-negative.

### Survival analyses

**Figure 1:**
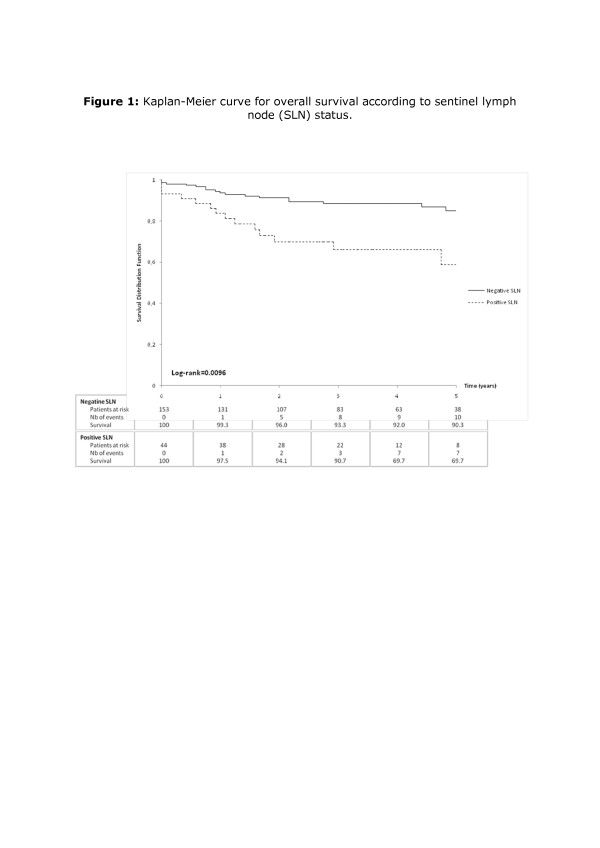
Kaplan-Meier curve for overall survival according to sentinel lymph node (SLN) status.

**Figure 2:**
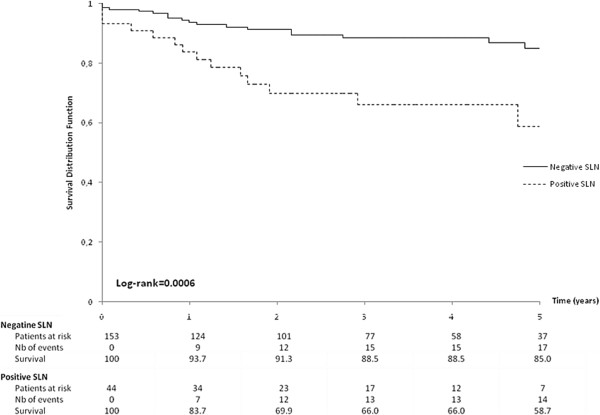
Kaplan-Meier curve for disease-free survival according to sentinel lymph node (SLN) status.

The overall cohort mortality rate was 11.3%. The mortality rate (Figure 1) was significantly higher in the SLN positive group than in the SLN negative group (20.4% versus 7.5%, p = 0.01). The 5-year overall survival (OS) rate was 84.6% for all patients, but was significantly higher for SLN negative patients as compared to SLN positive patients (90.3% versus 69.7%; p = 0.0096). The five-year disease-free survival (DFS) rate was 79.6% for all patients, but was significantly higher in SLN negative patients than in SLN positive patients (85.0% versus 58.7%; p = 0.0006) (Figure 2).

### Adverse events

Post-operative complications of SLN biopsy (neuropathic pain, infection, seroma, hematoma, lymphedema) were observed in 12% of patients (24/197). Three patients presented with severe complications such as cellulitis (2 patients) and severe invalidating complex regional pain syndrome occurred in one patient after a brachial plexus injury. Post-operative complications of additional CLND were observed in 14% of patients (6/42), including lymphedema (3), hematoma (1) and neuropathic pain (1) and complex regional pain syndrome (1).

## Discussion

Despite the small number of patients in our cohort, our results confirm previous studies on SLN analysis in melanoma [3,4,8-11], in terms of SLN identification rate (97%), percentage of SLN positive patients (22%) and percentage of additional positive CLND (25%). We also observed a significant association between positive SLN and primary tumor thickness and microscopic ulceration. Although only one SLN was harvested in 67% of our cases, the mean number of SLN harvested was 1.5 +/− 1 in our study, very similar to those found by previous studies [8,12]. Furthermore, Gershenwald 7. [9] found that among the 343 patients who underwent CLND, the majority (72%) had only one positive SLN as compared to 67% in our study. To our knowledge, there are neither consensus nor recommendations on the minimal or maximal number of SLN that should be harvested during the procedure.

As expected, recurrences were significantly more frequent in SLN positive patients (32%) than in SLN negative patients (13%) suggesting a better regional control of melanoma progression after SLNB and immediate CLND. The rate of locoregional lymph node relapses after lymph node excision in SLN positive patients varied between 0 and 20% [4,13]. Conversely, locoregional relapses after LND of clinically palpable lymph node palpation varied between 20 and 50% [13-15]. It is nonetheless still difficult to know whether better regional lymph node control is related to complementary CLND performed after positive SLN is discovered. Another study [13] retrospectively compared two groups of SLN positive patients from different hospitals, some of whom underwent additional CLND. The authors found no significant difference in terms of survival between the two groups. In our study, relapse and mortality rates in SLN positive patients were not influenced by the result of additional CLND casting further doubt on the benefits of complementary surgery.

The SLNB was considered false-negative if a primary recurrence developed in the regional lymph node basin from which a tumor-free SLN had been removed. In our study, the number of false-negative (10/153 = 6.5%) was similar (3-8%) to other studies [11,16,17]. However, there is ongoing debate on how to correctly calculate the false-negative rate. It should not be expressed as a percentage of the total population, but rather as the number of false-negative results divided by the number of false-negatives and true-positives [18]. In our study, the false negative rate (10/54 = 18.5%) was also similar to the rate calculated in other studies (5.7% to 21%) [14]. Beyond the technical problems associated with the SLN procedure, false negatives may be related to different factors: the time it takes to learn to perform the technique [19], lymphatic drainage disruption related to primary tumour excision, lymphatic obstruction by tumor cell embolism, the presence of a neck SLN [7], inadequate histological analysis and hematogenous dissemination.

The risk factors for recurrence after negative SLN are identical to those observed after positive SLN: presence or absence of macroscopic ulceration and tumoral thickness superior to 4 mm [7]. Analysis of all relapses (Table 3) according to SLN status (positive or negative) showed a poor sensitivity (41%) and positive predictive value (32%) for SLN analysis but good specificity (81%) and negative predictive value (87%). Although rarely calculated, these values are similar to those found by Saltman *et al.*[6] The same analysis focused on mortality confirmed the good specificity (80%) and negative predictive values (91%), i.e. similar to the findings of Morton *et al.*[4].

Our overall survival rates at 5 years (69.7% and 90.3% for SLN positive and negative patients, respectively) were similar to previous prognostic values of SLN analysis [8,20] when followed by additional CLND. A multivariate analysis of an international cohort of 2313 stage III patients [20] showed that the overall survival rate at 5 years was greater in patients presenting with micrometastases than in patients with palpable lymph node metastases (67% vs 43%). However, there were wide variations (23% to 87%) in patients with micrometastases depending on histological characteristics of primary melanoma (ulceration, mitotic index), its anatomical localization, number of SLN affected and patient age [20]. A bayesian analysis was recently carried out of studies with more than 50 patients undergoing SLN between 1993 and 2010 [11]. The authors focused on the prognostic benefit of the SLN analysis in terms of specific survival for melanoma with tumor thickness between 1 and 4 mm. For these patients, the risk of melanoma-related death varied between 26.2% and 31.6% when SLN was positive versus 9.7% and 15.6% when SLN was negative.

All of these results for relapse and survival involve the combination of two surgical procedures: research and analysis of SLN followed by additional CLND in the event of micrometastases in SLN. At the present time, no advantages of lymph node excision have been shown in terms of regional monitoring of metastatic damage or overall survival [13,15,21]. However, an additional lymph node excision is more often recommended when the SLN is invaded [22].

Many attempts have been made to predict non-SLN (NSLN) metastasis in the additional lymph node excision performed after positive SLN based on demographic, primary tumor, and SN features of patients with melanoma. Previous studies [9,10,23-25] indicate that overall SLN tumor burden, primary tumor thickness, and number of SLN harvested may be useful in identifying a group at low risk for positive NSLN. It is nonetheless interesting that these two parameters are also predictive for SLN micrometastases [20,26]. However, marked variability in the correlation of individual features with NSLN metastases and the degree of this correlation has characterized the literature on this issue to date [27,28]. There is currently no consensus regarding what degree of risk of NSLN involvement indicates that it is safe to forego CLND. Before elimination of CLND can be advocated, prospective clinical trials designed to assess the safety of omitting formal CLND with respect to survival and locoregional control in low-risk groups are needed. The ongoing Multicenter Selective Lymphadenectomy Trial II [29] which compares CLND versus close observation with sonography and clinical examination for patients with a positive SLN, should provide valuable information about which patients might be spared a CLND.

The frequency of post-operative complications (neuropathic pain, infection and lymphocele) observed in our study was similar to other studies in terms of morbidity related to SLN analysis [30].

## Conclusions

In conclusion, our retrospective study confirms the results of SLN analysis in patients with melanoma with tumor thickness greater than 1 mm. The main benefit of this analysis was the prognostic value in terms of relapse and survival (as long as additional lymph node excision was performed), and its high predictive negative value. The usefulness of complementary excision is still a subject of debate due to the high percentage of normal results and morbidity. Although already recommended, evaluating the benefit of additional CLND after positive SLN is necessary. New SLN analysis techniques are also under evaluation in order to lower operative morbidity [31].

## Competing interests

All authors have significantly contributed to the manuscript and all agree with its contents. All authors have read and approved the manuscript. Authors do not have any relevant financial interests in the findings from this manuscript.

## Authors’ contributions

CB-D, EP, DD, HB, FP, PH and FA, 1) have made substantial contributions to conception and design and acquisition, analysis and interpretation of data; 2) have been involved in drafting the manuscript or revising it critically for important intellectual content; and 3) have given final approval of the version to be published. MP and FS 1) have made substantial contributions to design and analysis and interpretation of data; 2) have been involved in revising the manuscript critically for important intellectual content; and 3) have given final approval of the version to be published. All authors read and approved the final manuscript.

## Pre-publication history

The pre-publication history for this paper can be accessed here:

http://www.biomedcentral.com/1471-5945/12/21/prepub
